# Production Optimization, Adjuvant Screening and Immunogenicity Evaluation of a Virus-like Vesicle Rabies Vaccine

**DOI:** 10.3390/vaccines13111122

**Published:** 2025-10-31

**Authors:** Xiaoyu Zhang, Xin Liu, Ying Wu, Zhenfang Fu, Ling Zhao, Ming Zhou

**Affiliations:** 1 National Key Laboratory of Agricultural Microbiology, Huazhong Agricultural University, Wuhan 430070, China; 17622890147@163.com (X.Z.); 15669581925@163.com (X.L.); 18845097685@163.com (Y.W.); zhenfu@uga.edu (Z.F.); 2 Key Laboratory of Preventive Veterinary Medicine of Hubei Province, College of Veterinary Medicine, Huazhong Agricultural University, Wuhan 430070, China; 3 Frontiers Science Center for Animal Breeding and Sustainable Production, Wuhan 430070, China; 4 Hubei Hongshan Laboratory, Wuhan 430070, China

**Keywords:** SFV-RVG, rabies vaccine, adjuvant optimization, robust immunogenicity, mouse and dog model

## Abstract

**Background/Objectives**: Rabies is a fatal zoonotic disease caused by the rabies virus (RABV), and effective therapeutic treatments are currently lacking. Vaccination remains the primary strategy for rabies control. The Semliki Forest virus-rabies virus glycoprotein (SFV-RVG), a virus-like vesicle rabies vaccine combining Semliki Forest virus replicase and rabies glycoprotein, has shown potential as a promising vaccine candidate. This study aimed to optimize the production of SFV-RVG and evaluate adjuvant formulations to improve its immunogenicity in both mice and dogs. **Methods**: SFV-RVG production was optimized by determining the optimal multiplicity of infection (MOI) at 0.03 and cell density at 1 × 10^6^–1.3 × 10^6^ cells/mL, followed by scaling up the process in bioreactors. Eleven adjuvant formulations were tested in mice and dogs to assess their effects on immunogenicity. Cytokine analysis and antibody responses were measured, including IFN-γ, IL-4, IgG2a/IgG1 ratios, and neutralizing antibody titers. **Results**: The optimized SFV-RVG production was successfully scaled up, and M103 adjuvant induced rapid early antibody titers in mice. In dogs, GEL02 led to the highest neutralizing antibody levels, exceeding 40 IU/mL by 28 days post-immunization. Cytokine analysis indicated that both M103 and GEL02 significantly enhanced IFN-γ and IL-4 expression, balancing the Th1/Th2 immune response. SFV-RVG with GEL02 demonstrated stronger immunogenicity than a commercial vaccine, and challenge studies confirmed robust protection against lethal RABV in mice. **Conclusions**: This study establishes GEL02 as a superior adjuvant for rabies vaccines and provides a scalable SFV-RVG production process. These findings highlight SFV-RVG with GEL02 as a promising rabies vaccine candidate for dogs, offering significant potential for rabies control.

## 1. Introduction

Rabies is an acute zoonotic disease caused by the rabies virus, which remains a significant public health threat due to its high mortality rate in both humans and animals [1]. Despite advances in medical treatment, the case fatality rate reaches nearly 100% once clinical symptoms manifest, leading to over 60,000 human deaths annually worldwide. In animals, particularly in wild populations, the disease is also fatal once symptoms appear. The rabies virus glycoprotein (G), localized on the surface of the virus, plays a crucial role in facilitating the production of virus-neutralizing antibodies (VNAs) [2].

While standardized vaccination protocols are capable of providing effective prevention, the currently authorized vaccines demonstrate inadequate immunogenicity, requiring multiple doses to achieve adequate levels of protection. This requirement for booster vaccinations significantly raises the costs associated with immunization. Conversely, live-attenuated vaccines, which are primarily used for wildlife, provide long-lasting immunity but pose risks of reversion to virulence [3]. These challenges highlight the pressing need for the development of innovative rabies vaccines that offer improved dose-sparing capabilities, enhanced safety profiles, and greater immunogenic potency. Virus-like vesicles (VLVs) are self-replicating, enveloped subunit vaccines that function as virus vectors. They are primarily composed of the replicase from the Semliki Forest virus (SFV) and the glycoprotein of the vesicular stomatitis virus (VSV-G). These VLVs are recognized for their safety and efficacy, making them a versatile and advanced platform for vaccine development [4]. To enhance their utility for rabies vaccination, VLVs have been specifically engineered to enable stable intracellular replication while simultaneously expressing the glycoprotein of the rabies virus (RABV-G). Through these modifications, evolved variants of VLVs have been shown to achieve impressive titers of 10^8^ focus-forming units per milliliter (FFU/mL).

The necessity for multi-dose vaccination schedules significantly increases the financial burden associated with immunization. Additionally, aluminum-based adjuvants have been shown to improve immune responses to both DNA vaccines and inactivated rabies vaccines. However, research has identified several limitations associated with aluminum hydroxide adjuvants, including the potential for reduced immune responses and various adverse effects, such as increased production of IgE, localized tissue damage, chronic inflammation, and neurotoxicity. Therefore, it is essential that adjuvants enhance the efficacy of protection against rabies within animal populations.

Dogs remain the primary source of rabies transmission in most endemic regions, making the development of safe and effective canine rabies vaccines crucial for rabies control [5].

This study aims to optimize the production efficiency of SFV-based VLVs and evaluate the use of various adjuvants for enhancing immune responses in animal models. Specifically, we seek to explore the potential of SFV-RVG VLVs, combined with GEL02 adjuvant, for inducing protective immunity against rabies in both mice and dogs.

## 2. Materials and Methods

### 2.1. Cells, Viral Strains, and Experimental Subjects

BHK-21 cells were maintained in a culture medium consisting of DMEM/RPMI 1640 (Gibco, Thermo Fisher Scientific, Waltham, MA, USA), supplemented with 10% fetal bovine serum (FBS; Fisher Scientific, Pittsburgh, PA, USA), 100 U/mL penicillin (Sigma-Aldrich, St. Louis, MO, USA), and 100 μg/mL streptomycin (Sigma-Aldrich, St. Louis, MO, USA). The cells were cultured at 37 °C in a humidified atmosphere containing 5% CO_2_ and passaged every 2–3 days to maintain optimal growth conditions.

Recombinant SFV-RVG constructs were generated and characterized in our laboratory. Viral stocks were amplified in BHK-21 cells, harvested, clarified by centrifugation, and stored at −80 °C until further use. The Challenge Virus Standard strain CVS-24, a highly pathogenic rabies virus strain, was employed for post-vaccination challenge assays to evaluate vaccine efficacy under lethal exposure conditions.

For animal studies, six-week-old female ICR mice were obtained from the Hubei Provincial Center for Disease Control and Prevention (Wuhan, China). The mice were acclimatized for one week before immunization and maintained under specific pathogen-free (SPF) conditions in the individually ventilated cage (IVC) facility of Huazhong Agricultural University. One-month-old Beagle dogs were purchased from Hubei Yizhicheng Biotechnology Co., Ltd. (Wuhan, Hubei, China) and housed at the Experimental Animal Center of Wuhan Keqian Biology Co., Ltd. (Wuhan, Hubei, China) The dogs were reared until three months of age before being randomly assigned into groups for immunization (*n* = 5 per group, with three females and two males in each group). All animals were provided ad libitum access to food and water, and were monitored daily for health and welfare.

### 2.2. Antibodies and Reagents

The monoclonal antibody (MAb) targeting the glycoprotein of the RABV, designated as clone 2B10, was developed and preserved within our laboratory for use in indirect immunofluorescence assays (IFA) and Western blot (WB) analyses. Secondary antibodies—specifically HRP-conjugated IgM, IgG, IgG1, and IgG2 antibodies, designed for use in enzyme-linked immunosorbent assays (ELISA) [6]—were obtained from Bethyl Laboratories in Montgomery, TX, USA. The tangential flow filtration (TFF) membrane cassette and core 400, utilized for virus concentration and purification, were acquired from Cobetter Consieve™ (Hangzhou, Zhejiang, China). These adjuvants were employed for the immunization of mice and dogs. Aluminum hydroxide gel was sourced from Invivogen Corporation (San Diego, CA, USA), while PoLyI: C [7], M101, M103 and M108 were supplied by Chengdu Iscon Biotech Co., Ltd. (Chengdu, Sichuan, China). MONTANIDETM GEL01 (GEL 01) [8], MONTANIDETM GEL02 (GEL 02) [9] and MONTANIDETM IMG 1313 VG N (IMS1313) were obtained from Seppic Inc. B-type CpG [10] and C-type CpG oligodeoxynucleotide mixtures were provided by Huapu Bioengineering (Nanjing, Jiangsu, China), while MS302 was sourced from Suzhou Womei Biotechnology Co., Ltd. (Suzhou, Jiangsu, China). The commercial inactivated rabies vaccine used was Nobivac^®^ Rabies (MSD Animal Health, Madison, NJ, USA) for dogs and cats, containing ≥2 IU of Pasteur RIV strain per dose and administered according to the manufacturer’s recommended protocol.

### 2.3. Investigations of Virus Propagation in Cell Culture and Shake Flask Systems

Thawed BHK-21 suspension cells were cultured in T25, T75, T175 flasks, and 125 mL shaker flasks. The cultures were initiated at varying seeding densities of 2.0 × 10^5^, 1.0 × 10^6^, and 1.3 × 10^6^ cells/mL, all samples were kept at a temperature of 37 °C and a pH level of 7.1. The cells were subsequently inoculated with SFV-RVG at MOIs of 0.01, 0.03, 0.05, and 0.1, followed by a culture period of four days. Sampling was conducted every 24 h to assess the viral growth curves corresponding to the different initial seeding densities. The optimal culture conditions were subsequently identified for the shaker flask cultures, with continued sampling every 24 h to monitor the viral growth curves at various seeding densities.

### 2.4. Infection and Proliferation of SFV-RVG in a Bioreactor System

In order to optimize SFV-RVG production, cultivations were performed in 5 L automated stirred-tank bioreactors using flat sheet carriers at a maximum loading of 50 g/L, following previously described procedures [11]. Bioreactors were calibrated for dissolved oxygen (DO), pH, and temperature prior to inoculation. Seed cultures with >95% viability and 4 × 10^6^ to 6 × 10^6^ cells/mL were used to initiate bioreactor cultivation. Cultures were maintained at 37 °C with DO at 50% air saturation and pH 7.2, using CO_2_ or NaHCO_3_ for pH control as needed [12]. Agitation was set at 100 rpm, and medium exchanges were performed during the stationary phase for SFV-RVG addition. Samples for metabolite and virus titer analyses were collected every 12 h, centrifuged to remove debris, and stored at −80 °C. Virus production in BHK-21 cells (MOI 0.03) and glucose monitoring were conducted as previously described.

### 2.5. Virus Ultrafiltration Concentration

Ultrafiltration concentration was performed using 0.11 m^2^ membrane cassettes following previously described protocols [13]. Membranes were integrity-tested and sanitized prior to use. The procedure included buffer equilibration, concentration, and ultrafiltration dialysis, with a final target volume achieved through controlled permeate removal and top wash steps. Two cassettes were used to retain ~150 mL of sample per run, and all tubing and system components were flushed with purified water before and after processing. Feed flow rates were maintained within recommended ranges to ensure optimal membrane performance.

### 2.6. Virus Chromatography Purification

The chromatography purification procedure commenced with the connection of a chromatography column, possessing a volume of 24 mL, to a peristaltic pump. The column was subsequently placed on a sterile operating table to ensure surface disinfection. Initially, the entire system was rinsed with 72 mL of ultrapure water at a flow rate of 10 mL/min to remove any residual ethanol [14]. Following this, the system underwent sanitization through the circulation of 1 mol/L NaOH at a pump speed of 10 revolutions per minute for a duration of one hour. After the sanitization process, the system was flushed with sterile water at a pump speed of 20 revolutions per minute. The system was then equilibrated using a sterile equilibration buffer at the same pump speed until the pH at the outlet reached 7.0. Subsequently, the concentrated virus feed was introduced into the column at a pump speed of 20 revolutions per minute. Sample collection commenced upon the initial rise in absorbance. The equilibrium liquid began to elute, and sample collection continued until the absorbance decreased to a value approaching zero, signifying the conclusion of the elution process.

### 2.7. SDS-PAGE and Western Blotting

SFV-RVG infected BHK-21 cells at MOI 0.03. Following a 48 h incubation period, Cell lysis was performed using RIPA buffer (Beyotime Biotechnology, Shanghai, China), after which the resulting lysates underwent 10% SDS-PAGE analysis. Protein transfer was performed onto 0.2 μm PVDF membranes (Merck Millipore, Burlington, MA, USA). Blocking with 5% skim milk/TBST (1 h, RT) was followed by overnight incubation with the anti-RABV-G monoclonal antibody. After the washes, secondary antibody was applied (45 min, RT). Following another three TBST washes, chemiluminescent detection was carried out using an HRP substrate (Merck Millipore, Burlington, MA, USA) to visualize protein bands.

### 2.8. Animal Immunization

#### 2.8.1. Immunization and Protection in Mice

A total of 130 six-week-old female ICR mice were randomly assigned to 13 groups. SFV-RVG vaccines were formulated with various adjuvants, including alum, PolyI:C, M101, M103, M108, GEL01, GEL02, IMS1313, B-type CpG, C-type CpG mixture, and MS302. Mice were immunized intramuscularly in the hind limbs, and blood samples were collected weekly for six weeks to determine VNA titers. The two adjuvants inducing the highest VNA levels were selected for further evaluation.

In a comparative immunogenicity and protective efficacy study, six-week-old female ICR mice (*n* = 10 per group) were immunized intramuscularly with inactivated SFV-RVG combined with either M103 or GEL02, or with formaldehyde-inactivated SFV-RVG (0.025% at 37 °C for 24 h), with DMEM as the negative control. Each injection was 100 μL. Mice were challenged intracerebrally with 50 LD_50_ of CVS-24 at 21 days post-immunization (dpi), and survival was monitored daily for 21 days. Moribund animals or those losing >25% body weight were euthanized according to established protocols [15].

#### 2.8.2. Immunization in Canines

All dogs enrolled in the study had no prior history of rabies vaccination, and pre-vaccination screening confirmed the absence of detectable VNAs in their sera. The animals were randomly assigned into three groups (*n* = 5 per group; 3 females and 2 males per group): (1) M103 group, receiving inactivated rabies antigen formulated with the M103 adjuvant; (2) GEL02 group, receiving the same antigen dose formulated with the GEL02 adjuvant; and (3) commercial vaccine control group, receiving a licensed commercial inactivated rabies vaccine. Each dog was administered a single subcutaneous dose containing 10^8^ FFU of antigen (or an equivalent dose of the commercial vaccine) in the dorsal neck region. Following vaccination, the animals were continuously monitored for one hour to detect any immediate adverse reactions and were subsequently examined at regular intervals. Blood samples were collected from the left cephalic vein at baseline (pre-vaccination) and at predetermined time points post-vaccination for the quantification of VNAs using a standard neutralization assay and for the assessment of cytokine responses (IFN-γ and IL-4) via commercial ELISA kits.

### 2.9. Titration of RABV-Specific VNA

The FAVN test measures the titers of rabies VNA [16]. Serum samples from mice and canines were subjected to inactivation through heating at 56 °C for a duration of 30 min. Following this procedure, DMEM (100 μL/well) was aliquoted into 96-well plates. The initial introduction of 50 μL of inactivated serum into column 1 was followed by a threefold serial dilution across the plate. Subsequent to the dilution process, the serum was subjected to incubation with 50 μL of CVS-11. containing 100 focus-forming units per well, at 37 °C for one hour to facilitate neutralization. Following this incubation, 100 μL of cells. After plating at 2 × 10^4^ cells/well, cultures underwent 72 h (37 °C, 5% CO_2_). BHK-21 were fixed with acetone that had been pre-cooled at −20 °C. Following a one-hour incubation at 37 °C utilizing FITC-labeled RABV-N mAb for cell staining, fluorescence was evaluated utilizing an Olympus IX51 microscope. The reference standards provided by the National Institute for Biological Standards and Control (NIBSC) located in Hertfordshire, UK, facilitated the normalization of emission intensity, allowing for the quantification of results in IU/mL.

### 2.10. ELISA for Determining RABV Specific Antibodies

Specific ELISAs for the RABV were conducted to evaluate the presence of various antibody isotypes. In brief, serum samples collected from mice were subjected to separation and inactivation at 56 °C for 30 min. Following this, purified rabies virus (RABV) virions at a concentration of 500 ng per well were utilized to coat the ELISA plates. The sample was diluted in a protein coating buffer composed of 5 mM sodium carbonate at a pH of 9.6 and incubated at 4 °C overnight and then use PBST (0.5% Tween 80) washed five times. after which they were blocked for two hours at 37 °C using a solution of 5% low-fat milk in PBS. Following the blocking procedure, 100 μL of diluted serum samples were introduced to each well, with dilutions of IgM (1:100), IgG (1:10,000), IgG1 (1:500), and IgG2 (1:1000). After blocking with 5% BSA, samples were incubated at 37 °C for 1 h and washed five times with PBST. Following this, 100 μL of horseradish peroxidase (HRP)-conjugated goat anti-mouse antibodies, which are specific to IgM, IgG, IgG1, or IgG2, was introduced to each well and allowed to incubate for 45 min at a temperature of 37 °C. TMB substrate (Biotime Biotechnology, Shanghai, China) was incubated, then reactions were quenched using SDS-containing solution. Measure the value at OD_630 nm_.

### 2.11. Detection of IFN-γ and IL-4

Supernatants from cell cultures, specifically mouse splenic lymphocytes at 28 dpi, were collected following approximately 72 h of stimulation with recombinant eukaryotic RABV-G protein. The levels of IFN-γ and IL-4 in these supernatants were assessed using commercial ELISA kits for Mouse IFN-γ and IL-4 (Multi Sciences, Hangzhou, Zhejiang, China), in alignment with the guidelines established by the manufacturer. The concentrations of the cytokines were determined based on the standard curve generated for each ELISA plate.

### 2.12. Statistical Examination

Statistical analyses were performed utilizing GraphPad Prism 10.0 software (GraphPad Software, San Diego, CA, USA). Results show mean ± SEM. Survival analysis used log-rank testing, while statistical analyses conducted to compare groups utilized one-way ANOVA.

### 2.13. Ethical Statement

The animal experiments were performed in compliance with the national laboratory animal guidelines set forth by the Ministry of Science and Technology of the People’s Republic of China. The Scientific Ethics Committee of Huazhong Agricultural University has granted approval for the study (Approval Nos. HZAUMO-2025-0170 and HZAUDO-2025-0004).

## 3. Results

### 3.1. Optimization of Cell Density, MOI, and Infection Duration for SFV-RVG Amplification

**Figure 1:**
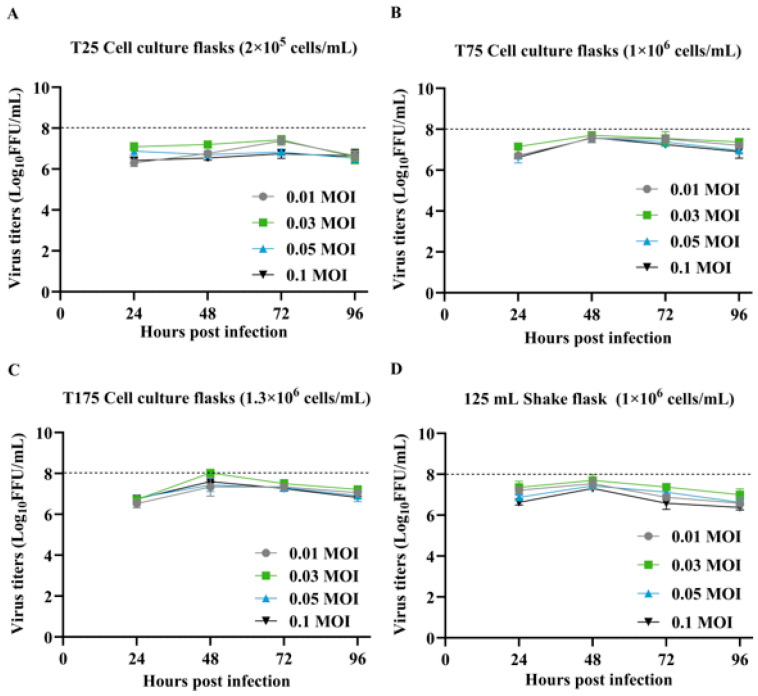
Growth kinetics of SFV-RVG in adhesion-adapted BHK-21 cells. (**A**–**D**) Dynamics of SFV-RVG virus titers produced in adhesion-adapted BHK-21 cells cultured in different vessels (T25, T75, T175 flasks, and 125 mL shake flask) at varying MOI. Data are derived from three independent experiments, each performed in triplicate. Error bars represent the SEM.

SFV-RVG has been constructed in our previous study, and we further investigated the production process of SFV-RVG in this study. Initially, the optimization for expansion culture of SFV-RVG is carried out. Several parameters, including cell density, MOI, and infection duration, can affect the proliferation of SFV-RVG. Therefore, we systematically investigated these parameters by seeding BHK-21 cells at approximately 2 × 10^5^, 1 × 10^6^, and 1.3 × 10^6^ cells/mL in T25, T75, and T175 culture flasks, and the cells were subsequently infected with SFV-RVG at MOI of 0.01, 0.03, 0.05, or 0.1, respectively. In order to perform viral titration, it is recommended that samples be collected at 24 h intervals from 24 h to 96 h following the infection. As shown in Figure 1A–C, the viral titers, regardless of different MOI, were peaked at 48 h post infection (hpi), indicating that the appropriate time point for virus collection is 48 hpi. Of note, among all tested cell densities, an MOI of 0.03 consistently yielded the highest titers of SFV-RVG, suggesting that MOI of 0.03 is the most appropriate condition. Moreover, as suspension cell culture systems can provide higher production, we then further investigated whether the optimal parameters for adherent cell culture are equally suitable for suspension cell culture system, and 125 mL shake flasks with a cell density of approximately 1.3 × 10^6^ cells/mL were then employed. As shown in Figure 1D, as expected, the MOI of 0.03, combined with harvesting at 48 hpi also achieved the highest virus titers in 125 mL shake flasks. Together, all these data suggest that the optimal production conditions for SFV-RVG are with a cell density of 1 × 10^6^–1.3 × 10^6^ cells/mL, an MOI of 0.03 and the harvest time at 48 hpi.

### 3.2. Large Scale Production of SFV-RVG in a Perfusion Bioreactor System

**Figure 2:**
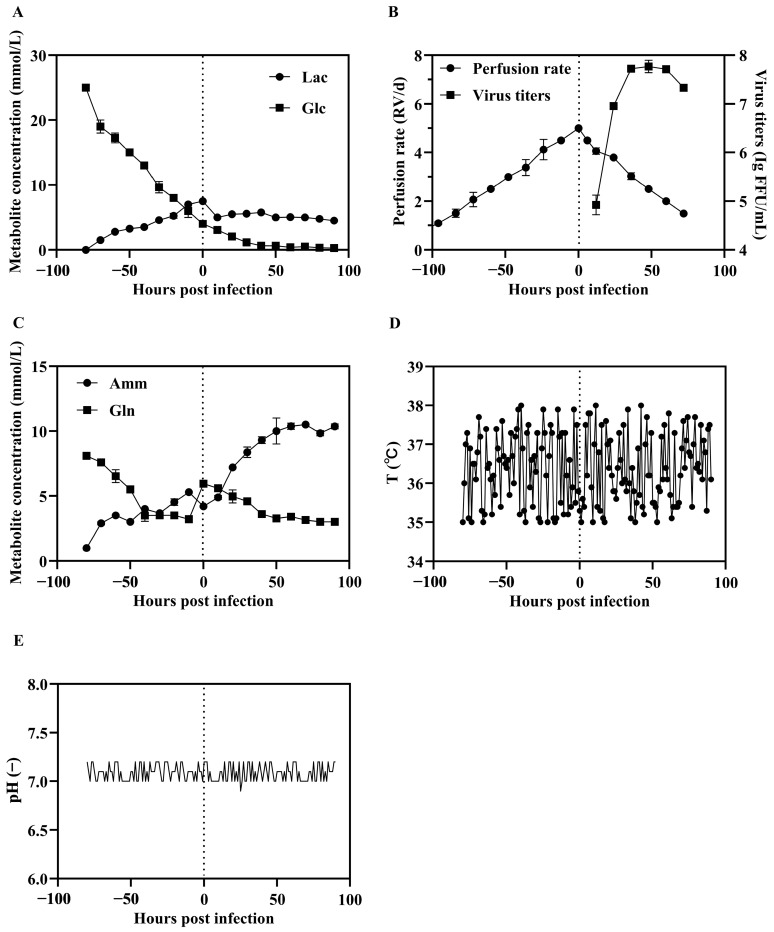
Illustrates the production kinetics of SFV-RVG in BHK-21 shake flask cultures. Panel (**A**) displays the levels of lactate (Lac) and glucose (Glc). Panel (**B**) depicts the viral titer alongside the perfusion rate. Panel (**C**) provides information on the concentrations of ammonium (Amm) and glutamine (Gln). Panel (**D**) outlines the temperature profile, while Panel (**E**) indicates fluctuations in pH. Vertical dotted lines signify the timing of infection. Error bars represent standard deviations derived from duplicate experiments.

Perfusion bioreactors operate in a continuous mode, where fresh media is continuously fed into the culture system while spent medium is removed, and cells are retained within the system via a cell retention device. This setup allows BHK-21 cells to be maintained under optimal conditions for prolonged periods. Throughout the perfusion, we monitored key biochemical parameters to the cells remaining in an optimal physiological state prior to SFV-RVG infection. One of the critical factors was glucose concentration. We set the glucose concentrations in culture medium in a range of 1.40 to 3.61 g/L (maintaining levels above 1.4 g/L) to ensure adequate nutrient availability for cell growth. Additionally, the pH was controlled between 7.0 and 7.2. By taking samples at 12 h intervals, we were able to adjust the perfusion rate and nutrient supplementation to meet the dynamic metabolic demands of the culture. Monitoring glucose consumption and plotting its depletion curve allowed us to identify when the BHK-21 cells began approaching a plateau phase, which occurred by 72 h (Figure 2A). Accordingly, we performed the medium change at 60 h, when the cells remained in the exponential growth phase at a density of 1.3 × 10^6^ cells/mL. After replacing the spent medium with fresh medium, we initiated SFV-RVG infection at an MOI of 0.03, aligning with our prior small-scale optimizations. Drawing on prior small-scale optimization in flasks and shake flasks, we employed an MOI of 0.03 for the perfusion-based bioreactor process. Titer assays were subsequently conducted to evaluate virus production levels, demonstrating that SFV-RVG proliferation under these conditions in the perfusion bioreactor system.

Above all, these results underscore the advantages of perfusion-based cultivation in supporting cell growth and virus proliferation, which provides a stable, scalable bioreactor process for large scale production of SFV-RVG vaccine.

### 3.3. Purification and Verification of the Expression of G Protein by SFV-RVG

**Figure 3:**
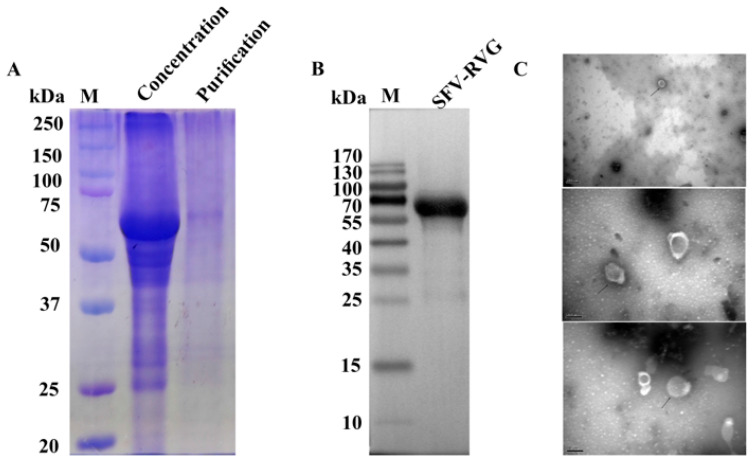
Detection of RABV-G by SDS-PAGE and Western blot assays. (**A**) SDS-PAGE (**B**) Western blot (**C**) Purified SFV-RVG virions (TEM, a–c) versus size-varied fixed/stained VLVs.

To verify whether SFV-RVG with large scale production can successfully express RVG after concentration and purification, a membrane-based filtration approach was employed to concentrate the SFV-RVG protein, followed by column chromatography for purification. The effectiveness of the purification process was assessed by Coomassie Brilliant Blue staining. As shown in Figure 3A, the concentrated SFV-RVG, prior to column chromatography, contained multiple contaminating proteins ranging from 50 to 75 kDa, while the SFV-RVG band at approximately 65 kDa (corresponding to the RVG) became almost the only observed band after column chromatography, indicating the success of purification (Figure 3A). Furthermore, the expression of RVG by purified SFV-RVG was also determined by Western blot analysis, and the result suggested that the RVG was well expressed by purified SFV-RVG with large scale production. Additionally, as the viron integrity of SFV-RVG after large scale production and purification is an important factor that affect the vaccine immunogenicity of SFV-RVG, a transmission electron microscopy was then carried out to observe the morphology of purified SFV-RVG that produced by the perfusion bioreactor system. As shown in Figure 3C, different sizes of purified SFV-RVG were observed with an intact particle shape, and the sizes ranged from 52 to 132 nm, with a mean size of 98 nm, indicating that the virions of SFV-RVG with large scale production are still intact, which is similar with that produced with adherent cell culture system.

### 3.4. Adjuvant Selection and Immunogenicity Evaluation of Adjuvanted SFV-RVG Vaccine in Mice

**Figure 4:**
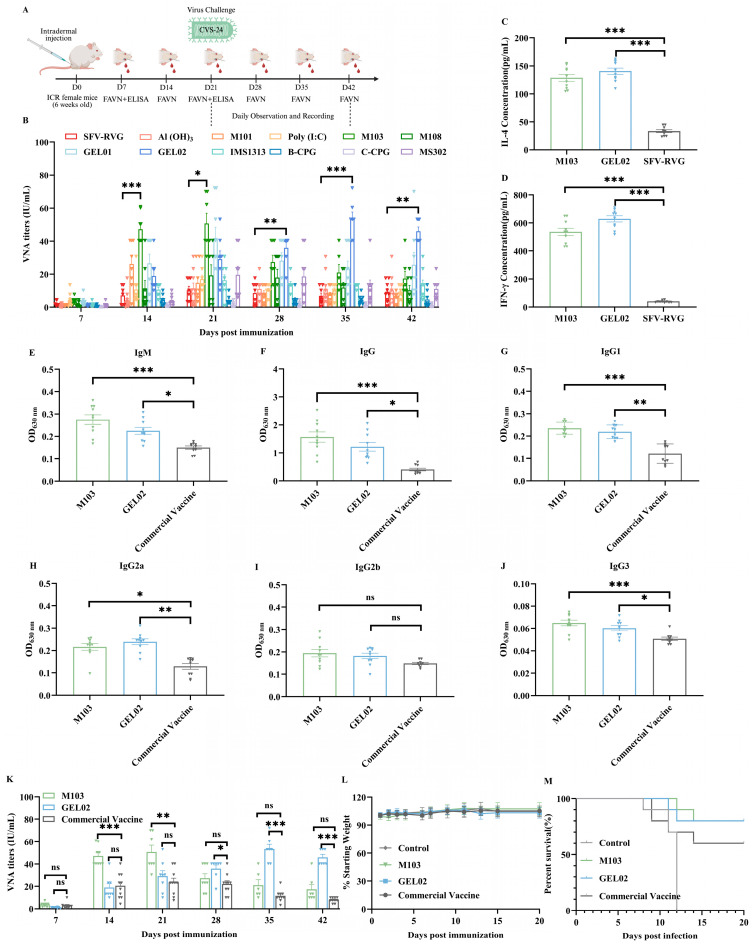
Illustrates the profiles of humoral and cellular immunity in ICR mice that were immunized with adjuvanted SFV-RVG vaccines. (**A**) The immunization schedule indicates a prime dose administered at week 0, accompanied by weekly serum sampling. (**B**,**K**) The titers of rabies virus-neutralizing antibodies (VNA) were assessed using the FAVN assay, with sample sizes of *n* = 10 for panel B and *n* = 5 for panel K. (**C**–**H**) The kinetics of serum immunoglobulins were evaluated through indirect ELISA on days 7 and 21, measuring IgM (**C**), IgG (**D**), IgG1 (**E**), IgG2a (**F**), IgG2b (**G**), and IgG3 (**H**). (**I**,**J**) The secretion of cytokines (IL-4 and IFN-γ) in splenocyte cultures was quantified using ELISA. (**L**,**M**) Changes in body weight and survival curves were also recorded. Vertical dotted lines indicate the time points of infection. Error bars represent the SEM; * *p* < 0.05, ** *p* < 0.01, *** *p* < 0.001, and ns indicates non-significant results.

To enhance the immunogenicity of SFV-RVG, eleven different adjuvants were selected to combined with SFV-RVG, including Aluminum Hydroxide, M101, PloyI: C, M103, M108, GEL01, GEL02, IMS1313, B-CPG, C-CPG, MS302, and female ICR mice (*n* = 10/group) were intramuscularly immunized with SFV-RVG vaccine containing one of the above adjuvants. Blood samples were collected at 7, 14, 21, 28, 35, 42 dpi for VNA tests by FAVN assay, and the experiment design flow chart was as shown in Figure 4A. The results showed that all adjuvanted SFV-RVG elicited or sustained higher VNA levels compared to SFV-RVG alone at each tested time points, with M103 and GEL02 producing the most pronounced responses. Specifically, the M103 group induced the highest VNA titers at both 14 and 21 dpi, and began to drop down from 28 dpi, while GEL02 group showed the highest VNA titers from 28 dpi to 42 dpi as shown in Figure 4B. All the above data indicated that M103 could provide the fastest immune responses at early stage while GEL02 showed the highest long lasting VNA titers (at least for 42 days). In addition, the levels of IFN-γ and IL-4 that as indexes for Th1 and Th2 cellular immunity, respectively, were significantly higher in the M103 and GEL02 adjuvant groups than those in SFV-RVG group as shown in Figure 4C,D, indicating that the two adjuvants can induce robust and balanced cellular immune responses. Therefore, the two adjuvants were then chosen as the most effective adjuvants for SFV-RVG. The immunogenicity of M103 or GEL02 adjuvanted SFV-RVG were then compared with a commercial rabies vaccine in a mouse model via intramuscular immunization route, and the levels of RABV-G specific total IgM, total IgG, IgG1, IgG2a, IgG2b, and IgG3 were then evaluated with ELISA at 21 dpi. The results showed that the levels of all determined RABV-G specific antibodies in M103 or GEL02 adjuvanted SFV-RVG immunized mice were significantly higher than those in commercial vaccine immunized mice (Figure 4E–G, respectively). For the VNA levels, the M103 adjuvanted SFV-RVG induced significantly higher levels than commercial vaccine at 14 and 21 dpi, while GEL02 adjuvanted SFV-RVG induced significantly higher levels of VNA than those in commercial vaccine group at 28, 35 and 42 dpi as shown in Figure 4K. Moreover, no clinical symptoms or body weight changes after immunization with each vaccine were observed as shown in Figure 4L, indicating that SFV-RVG adjuvanted with M103 or GEL02 is safe after immunization as the commercial vaccine. Meanwhile, to compare the efficacy of M103 or GEL02 adjuvanted SFV-RVG with commercial vaccine, the immunized mice were challenged with 50 times the LD_50_ of CVS-24, and the survival rates were analyzed. Notably, the non-immunized mice were all died between 8 and 12 days after challenge, and the commercial vaccine provided a survival rate of 60%. In contrast, the GEL02 or M103 adjuvanted SFV-RVG provided the same survival rate of 80%. All these data suggest that the adjuvant GEL02 and M103 could enhance the immunogenicity of SFV-RVG and provide a better protection against lethal RABV challenge in mouse model.

### 3.5. Immunogenicity Evaluation of Adjuvanted SFV-RVG Vaccines in Dogs

**Figure 5:**
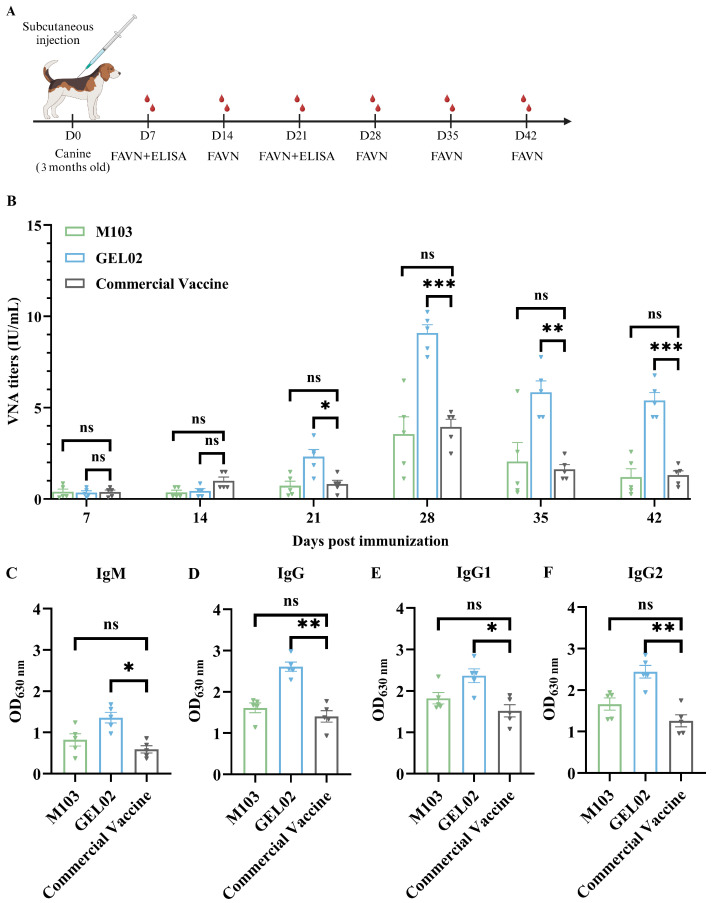
Illustrates the humoral immune responses observed in canines that were immunized with adjuvant-formulated SFV-RVG vaccines. Panel (**A**) outlines the experimental timeline, which includes the initial immunization (week 0) followed by weekly serum collections. Panel (**B**) presents the rabies VNA titers as determined by the FAVN assay (*n* = 5). Panels (**C**–**F**) display the serum titers of IgM (**C**), IgG (**D**), IgG1 (**E**), and IgG2 (**F**) as measured by indirect ELISA at days 7 and 21 post-immunization. Error bars: SEM. * *p* < 0.05, ** *p* < 0.01, *** *p* < 0.001, and ns indicates non-significant results.

To further evaluate the efficacy of adjuvanted SFV-RVG in dogs, a total of 25 three-month-old dogs were randomly divided into five groups (*n* = 5 per group) and subcutaneously immunized with SFV-RVG adjuvanted with M103 or GEL02, or a commercial vaccine, and the blood samples were collected on 7, 14, 21, 28, 35, and 42 dpi to determine the VNA titers with FAVN assay (Figure 5A). As shown in Figure 5B, a significantly higher VNA titers were observed in dogs immunized with GEL02 adjuvanted SFV-RVG vaccine than those in dogs immunized with M103 adjuvanted SFV-RVG or the commercial vaccine at 21, 28, 35, and 42 dpi. Consistently, the levels of IgM, total IgG, IgG1, and IgG2 in dogs immunized with GEL02 adjuvanted SFV-RVG were significantly higher than those in dogs immunized with M103 adjuvanted SFV-RVG or the commercial vaccine as shown in Figure 5C–F, respectively, demonstrating that M103, rather than GEL02, adjuvanted SFV-RVG provided a better immune response in dog model.

In summary, our study demonstrates that SFV-RVG can be optimized to high-yield manufacturing, and the immunogenicity of SFV-RVG in dogs could be significantly improved with GEL02 adjuvant, indicating that GEL02 adjuvanted SFV-RVG could be a promising rabies vaccine candidate for dogs with higher safety and immunogenicity.

## 4. Discussion

Rabies is an old zoonotic disease with nearly 100% fatality, and it still represents a severe public health threat all over the world. Although no effective treatment exists, rabies can be prevented through timely vaccination, which remains the cornerstone of disease control.

In our previous work, we constructed a virus-like vesicle expressing SFV-RVG [3], which demonstrated strong immunogenicity in mice, suggesting its potential as a candidate rabies vaccine. However, its applicability and performance in canine models have not been fully elucidated.

In the present study, we first optimized viral production parameters-multiplicity of infection, infection timing, and cell density-to enhance the scalability of VLV manufacturing under conditions that mimic industrial bioreactor systems [17]. Subsequently, we evaluated the immunogenicity of the SFV-RVG VLV formulated with two distinct adjuvants, M103 and GEL02, in mice and dogs.

In mice, both M103 and GEL02 markedly enhanced humoral and cellular immune responses compared with the non-adjuvanted group, with M103 inducing earlier and stronger VNA production and higher IFN-γ and IL-4 levels, indicating balanced Th1/Th2 activation. In contrast, in dogs, GEL02-adjuvanted formulations elicited higher and more sustained neutralizing antibody titers than M103, suggesting superior humoral immunogenicity. These findings indicate that while M103 is more effective in promoting early and balanced immune activation in mice, GEL02 may better support long-term antibody persistence in dogs.

Our observations are consistent with previous reports that adjuvant performance can vary markedly between species, likely due to differences in innate immune signaling pathways and antigen–adjuvant interactions [18,19,20,21]. This highlights the necessity of species-specific evaluation when developing veterinary vaccines. Nevertheless, the mechanisms underlying these species-dependent effects require further investigation.

Several limitations should be noted. Viral concentration and purification involved trade-offs between antigen yield and impurity removal [22]. Additionally, cellular immunity was directly assessed only in mice, while canine cellular responses were not directly measured and should be interpreted with caution. Furthermore, the experiments were conducted under controlled laboratory conditions that may not fully reflect vaccine efficacy under field circumstances [23]. Future work will focus on optimizing purification procedures to maximize antigen recovery, performing comprehensive analyses of both antibody and T-cell responses across species, and evaluating vaccine performance under real-world conditions [24].

## 5. Conclusions

Our study found that high-yield manufactured SFV-RVG with GEL02 adjuvant can enhance the immunogenicity and facilitated a balanced Th1/Th2 immune response in mouse and dog models, which delineates a strategic framework that significantly enhances the translational potential of SFV-RVG virus-like vesicles vaccine for a highly effective dog rabies vaccine.

## Data Availability

The data generated and analyzed during the current study are available from the corresponding author upon reasonable request.

## Abbreviations

The following abbreviations are used in this manuscript:

RABV	rabies virus
MOI	multiplicity of infection
VNAs	virus-neutralizing antibodies
VSV	vesicular stomatitis virus
TOI	time of infection
CCI	cell concentration at infection
MAb	monoclonal antibody
IFA	immunofluorescence assays
WB	Western blot
TFF	tangential flow filtration
DO	dissolved oxygen
Lac	lactate
Glc	glucose
Amm	ammonium
Gln	glutamine
